# Search for signals of positive selection of circadian rhythm genes PER1, PER2, PER3 in different human populations

**DOI:** 10.18699/vjgb-24-71

**Published:** 2024-10

**Authors:** A.I. Mishina, S.Y. Bakoev, A.Y. Oorzhak, A.A. Keskinov, Sh.Sh. Kabieva, A.V. Korobeinikova, V.S. Yudin, M.M. Bobrova, D.A. Shestakov, V.V . Makarov, L.V. Getmantseva

**Affiliations:** Centre for Strategic Planning and Management of Biomedical Health Risks of the Federal Medical Biological Agency, Moscow, Russia; Centre for Strategic Planning and Management of Biomedical Health Risks of the Federal Medical Biological Agency, Moscow, Russia; Centre for Strategic Planning and Management of Biomedical Health Risks of the Federal Medical Biological Agency, Moscow, Russia; Centre for Strategic Planning and Management of Biomedical Health Risks of the Federal Medical Biological Agency, Moscow, Russia; Centre for Strategic Planning and Management of Biomedical Health Risks of the Federal Medical Biological Agency, Moscow, Russia; Centre for Strategic Planning and Management of Biomedical Health Risks of the Federal Medical Biological Agency, Moscow, Russia; Centre for Strategic Planning and Management of Biomedical Health Risks of the Federal Medical Biological Agency, Moscow, Russia; Centre for Strategic Planning and Management of Biomedical Health Risks of the Federal Medical Biological Agency, Moscow, Russia; Centre for Strategic Planning and Management of Biomedical Health Risks of the Federal Medical Biological Agency, Moscow, Russia; Centre for Strategic Planning and Management of Biomedical Health Risks of the Federal Medical Biological Agency, Moscow, Russia; Centre for Strategic Planning and Management of Biomedical Health Risks of the Federal Medical Biological Agency, Moscow, Russia

**Keywords:** populations, SNP, adaptation, PER1, PER2, PER3, популяции, SNP, адаптация, PER1, PER2, PER3

## Abstract

The diversity of geographically distributed human populations shows considerable variation in external and internal traits of individuals. Such differences are largely attributed to genetic adaptation to various environmental influences, which include changes in climatic conditions, variations in sleep and wakefulness, dietary variations, and others. Whole-genome data from individuals of different populations make it possible to determine the specific genetic sites responsible for adaptations and to further understand the genetic structure underlying human adaptive characteristics. In this article, we searched for signals of single nucleotide polymorphisms (SNPs) under selection pressure in people of different populations. To identify selection signals in different population groups, the PER1, PER2 and PER3 genes that are involved in the coordination of thermogenic functions and regulation of circadian rhythms, which is directly reflected in the adaptive abilities of the organism, were investigated. Data were analyzed using publicly available data from the 1000 Genomes Project for 23 populations. The Extended Haplotype Homozygosity Score statistical method was chosen to search for traces of selection. The comparative analysis performed identified points subject to selection pressure. The SNPs were annotated through the GWAS catalog and manually by analyzing Internet resources. This study suggests that living conditions, climate, and other external factors directly influence the genetic structure of populations and vary across races and geographic locations. In addition, many of the selection variants in the PER1, PER2, PER3 genes appear to regulate biological processes that are associated with major modern diseases, including obesity, cancer, metabolic syndrome, bipolar personality disorder, depression, rheumatoid arthritis, diabetes mellitus, lupus erythematosus, stroke and Alzheimer’s disease, making them extremely interesting targets for further research aimed at identifying the genetic causes of human disease.

## Abstract (ru)

Разнообразие географически распределенных человеческих популяций демонстрирует большую вариацию внешних и внутренних признаков индивидов. Такие различия в значительной степени объясняются генетической адаптацией к различным воздействиям окружающей среды, к которым относят изменения климатических условий, колебания условий сна и бодрствования, вариации рациона и другие. Полногеномные данные, полученные от людей различных популяций, дают возможность идентифицировать конкретные генетические участки, ответственные за эти адаптации, и глубже понимать генетическую структуру, лежащую в основе адаптивных характеристик человека. В данной работе проведен поиск сигналов однонуклеотидных полиморфизмов (SNP), находящихся под давлением отбора у людей различных популяций. Для выявления сигналов отбора в различных популяционных группах были исследованы гены PER1, PER2 и PER3, играющие важнейшую роль в координации термогенных функций и регуляции циркадных ритмов, что напрямую отражается на адаптационных способностях организма. Анализ данных осуществляли на основе общедоступных данных из проекта «1000 геномов» (1000 Genomes Project) по 23 популяциям. Для поиска следов отбора был выбран статистический метод XP-EHH (expanded haplotype homozygosity score). Проведенный сравнительный анализ позволил идентифицировать точки, подверженные давлению отбора. Найденные SNP были аннотированы через каталог GWAS, а также вручную, путем анализа интернет-ресурсов и публикаций. Исследование позволяет сделать вывод о том, что условия проживания, климат и другие внешние факторы напрямую влияют на генетическую структуру популяций и варьируют в зависимости от расы и географического местоположения. Кроме того, многие из вариантов отбора в генах PER1, PER2, PER3, по-видимому, регулируют биологические процессы, связанные с основными современными заболеваниями, включая ожирение, онкологию, метаболический синдром, биполярное расстройство личности, депрессию, ревматоидный артрит, сахарный диабет, красную волчанку, инсульт и болезнь Альцгеймера, что делает их крайне интересными объектами для дальнейших исследований, направленных на идентификацию генетически обусловленных причин заболеваний человека.

## Introduction

Advances in SNP genotyping methods have led to a rapid shift from studies focused on spatially explicit neutral genetic processes to those focused on adaptive genetic processes (Ahrens et al., 2018). One tool for tracking these processes is the search of loci under selection pressure (Carlson et al., 2005). Unique genetic patterns or traces left in genomic regions subjected to selection are called selection signatures (Nielsen, 2005; Jensen et al., 2016; Bakoev et al., 2021). Selection signatures are genomic regions containing DNA sequences functionally involved in the genetic variability of the traits subject to selection (Lopez et al., 2015; Bakoev et al., 2023). Such parts are of interest because of their relevance for tracing evolutionary biology and potential links to genes that control phenotypes in wild and domestic populations (Xu et al., 2015).

Various statistical approaches have been used to identify loci under selection pressure, one of them being extended haplotype homozygosity (EHH) analysis. It should be noted that the word “homozygosity”, as part of the term EHH, refers to the probability that two randomly selected chromosomes from a population are identical (at a particular locus or region) (Klassmann, Gautier, 2022). The result interpreted from the theory is that major haplotypes with unusually high EHH and high population frequency indicate the presence of a mutation that became prominent in the human gene pool faster than expected under neutral evolution (Sabeti et al., 2002).

To study the genetic diversity and evolution of human populations, the XP-EHH (Extended Haplotype Homozygosity Score) method is well established to identify potential sites of genetic variation that may be associated with adaptation to different environments and conditions (Voight et al., 2006)

The PER1, PER2 and PER3 genes are involved in the coordination of circadian rhythms, regulation of the body’s adaptive abilities, and are also associated with various diseases (Liberman et al., 2017; Rijo-Ferreira, Takahashi, 2019). For example, a study found a high association of PER2 gene expression with the adaptation of organisms to low temperatures. S. Chappuis and co-authors (Chappuis et al., 2013) proved that mice with the Period2 (PER2) gene turned off are sensitive to cold because their adaptive thermogenesis system becomes less efficient. Regarding the PER1 gene, Y. Shi et al. (2021) claim that light adaptation generated by the CRTC1- SIK1 pathway, in which the PER1 gene is involved, in the suprachiasmatic nucleus provides a robust mechanism that allows the circadian system to maintain homeostasis in the presence of light perturbations. This mechanism appears to be important for rapid adaptation to changing environmental conditions. According to the findings of L. Zhang et al. (2013), a polymorphism in the PER3 gene is associated with the level of adaptation to shift work schedules and alternating sleep phases in nurses working in shifts.

Thus, genes from the PER group are a promising target for finding signals of positive selection in different human populations. In addition, the existence of a link between adaptive abilities, selection signals and major modern diseases is of interest.

## Materials and methods

Public data from The 1000 Genomes Project Consortium (1000 Genomes, 2008) representing 23 populations grouped into their respective clusters were used for analysis (see the Table).

**Table 1.**
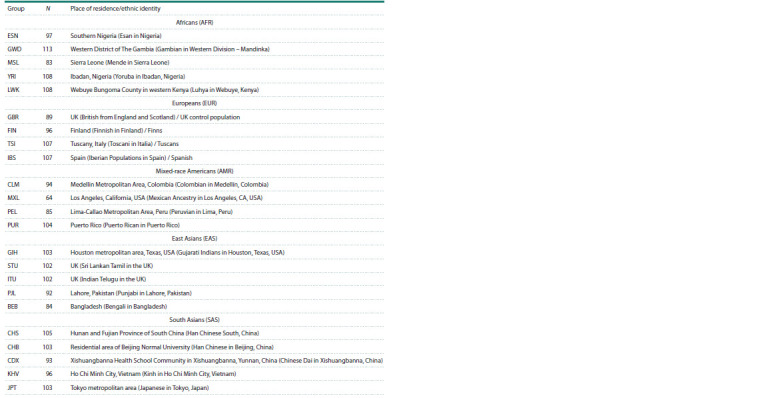
Populations from the 1000 Genomes Project selected for analysis

Plink 1.9 (Purcell et al., 2007) was used to merge all data. Using bcftools, we removed SNP duplicates and SNPs with identical positions, and normalized all data according to the GRCh38 reference. Start and end positions for the PER1, PER2, and PER3 gene regions (GRCh 38 assembly) were obtained from NCBI (National Library of Medicine (USA)).

The XP-EHH (Extended Haplotype Homozygosity Score) method implemented in the selscan program (Szpiech, 2021) was used to identify selection signals. Non-standardized scores were normalized using the “norm” script provided in the selscan program. SNPs with values crit = 1/–1 were considered as genetic variants under selection pressure (outliers) (crit = 1 – ancestral allele under selection pressure, crit = –1 – derived allele).

Selection signals were determined by inter-population comparisons, using YRIs from the African cluster as the comparison group. This allowed us to determine the outliers between the Yoruba African population (YRI) from Ibadan and other groups (including the African cluster, namely ESN, GWD, MSL and LWK). In addition, selection choices related to within-cluster variability were also of interest. For this purpose, a comparison group was selected in each cluster and analyzed with other groups in the same cluster. Thus, in the EUR cluster, GBR was taken as the comparison group and accordingly analyzed between GBR&FIN, GBR&IBS and GBR&TSI. In the AMR cluster, the PUR group was taken and analyzed between PUR&CLM, PUR&MXL and PUR&PEL. In the EAS and SAS clusters, CHB and BEB groups were defined, respectively, and analyzed between CHB&CDX, CHB&CHS, CHB&KHV, CHB&JPT and BEB&PJL, BEB&ITU, BEB&STU, BEB&GIH, respectively

## Results and their discussion

Genomics and molecular biology have strongly influenced research on “selection and adaptation” through the identification of the genetic basis of various traits associated with maintenance and health in humans and animals (Hancock et al., 2010; Gintis et al., 2012). Alongside this, the results of the “genetic and genomic revolution” have enabled genome sequencing and provided new tools to measure both past and possibly ongoing adaptations (Zheng et al., 2023).

Human behavior is assumed to be determined by the interaction between nature and societal development (Saravanan et al., 2020). It can be assumed that the features of genetic structure in different human populations, including those associated with the exit of people from Africa, further formed the basis of individual features of human development (Benton et al., 2021). Thus, the PER1, PER2, and PER3 genes we considered showed signals of positive selection, some of which were seen in several populations (these variants are mainly localized in the PER2 gene), while others were found in only one population.

**Fig. 1.**
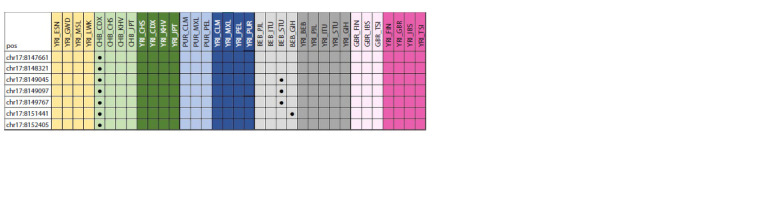
Genetic variants under selection pressure in the PER1 gene. Here and in Fig. 2 and 3 pos – position.

Analysis of the full-genome profiles of the studied populations revealed 110 loci (78 points in the PER2 gene, 25 in PER3 and 7 in PER1) under selection pressure. When analyzing the PER1 gene, eight outliers were detected in an intergroup comparison of South Asians living in Beijing (CHB) with South Asians living in Yunan (CDX) (Fig. 1). In addition, four selection pressure sites were also identified in an intergroup comparison of East Asians living in Bangladesh (BEB) with East Asians living in Sri Lanka (STU) and East Asians living in Bangladesh (BEB) with East Asians living in Houston, Texas, Gujarat (GIH) (Fig. 1).

The sites discovered are involved in processes such as: predisposition to the development of major depressive disorder, Parkinson’s disease, Alzheimer’s disease, alcohol addiction, and breast cancer, as well as longevity (see Supplementary Material)1.


Supplementary Materials are available in the online version of the paper: https://vavilov.elpub.ru/jour/manager/files/Suppl_Mishina_Engl_28_6.pdf


We would like to pay attention to points under selection pressure in several of the compared groups. Such SNPs were identified in an intergroup comparison of East and South Asians. The points found suggest that similar external factors acted on the compared groups, which had the same effect necessary for the adaptation of the ethnic groups under study. Significant signals at positions chr17:8149097 (predisposition to breast cancer) are worth noting. It is possible that the fixation of alleles in the comparison groups of East Asians from Bangladesh (BEB) and East Asians from Srilanka (STU), and South Asians from Beijing (CHB) and South Asians from Yunnan (CDX) could have occurred due to the prevalence of humid climate in the territories where the ethnicities studied lived. According to some authors, humid climate may be a risk factor in the development of a number of cancers (Maryanaji, 2020; Guo et al., 2021; Pan et al., 2023).

**Fig. 2.**
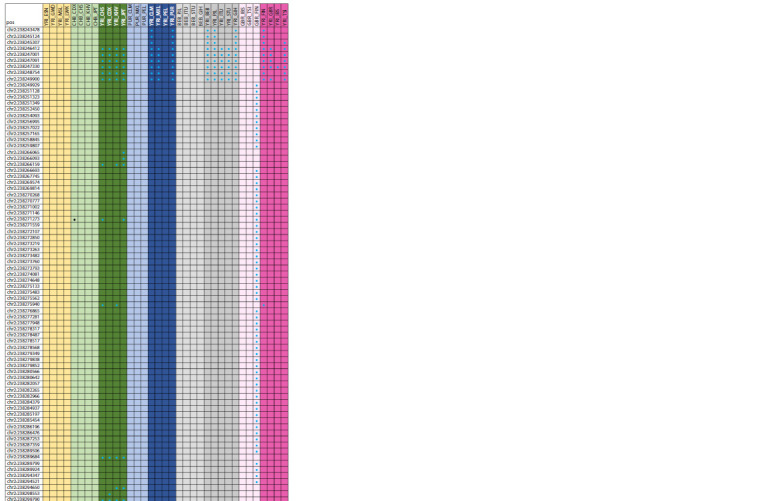
Genetic variants under selection pressure in the PER2 gene. Dots represent variants under selection pressure; black color of the dots means that the ancestral allele was under selection pressure, blue color stands for the derived allele.

In the analysis of the PER2 gene, 78 points under selection pressure were identified. Outliers were identified while comparing all of the ethnicities studied with Africans and in single sites in the intergroup comparison of South Asians and Europeans (Fig. 2). Analyzing the points in the compared population groups, it can be concluded that the African population is strongly differentiated from the other ethnicities studied. Annotation of sites under selection pressure in several of the compared groups revealed SNP involvement in the formation of chronotypes, sleep coordination, predisposition to diabetes, stroke, lupus erythematosus and bipolar disorder, intestinal cholesterol absorption, and associations with metabolic phenotype. The associations of SNPs with various diseases and phenotypes in humans are presented in more detail in Supplementary Material.

The presence of the total number of outliers when comparing the population group of Africans and other ethnic groups indicates a long period of influence of certain external factors on all the studied populations. It is interesting to note that all alleles under selection pressure turned out to be derived variants. Since the leading function of the PER2 gene is the formation of chronotypes, it can be assumed that the finding of the total array of points under selection pressure is also explained by the action of external factors inherent in the area where the studied ethnic groups lived. Such factors include the total number of daylight hours, magnetic field action, climatic peculiarities and others

Identification of loci under selection pressure in several compared groups between South Asians and Africans, as well as within the groups of South Asians living in Beijing (CHB) and Yunnan (CDX) revealed SNPs responsible for predisposition to the development of a number of gastrointestinal and cardiovascular diseases. At the same time, derived alleles are identified in groups comparing South Asians with Africans, and when comparing South Asians within subgroups based on the region of residence, an ancestral allele is detected. Most interesting is the outlier in chr2:238289684 that was found when comparing South Asians to Africans: it is associated with systemic lupus erythematosus, which is caused by disorders such as hormonal imbalance during puberty, stress, and environmental factors, namely sun exposure and viral infections (Quaglia et al., 2021; Kim et al., 2022; Molina et al., 2022).

In our opinion, different levels of viral load as well as authentic climatic conditions may have played a key role in the development of adaptive abilities of these ethnic groups, thus fixing these alleles in the studied populations. The same theory may explain the fixation of loci associated with gastrointestinal diseases. As people migrated from the African continent to other areas, their gastronomic preferences changed, thus modifying the gut microbial ecosystem (Clemente et al., 2015; Syromyatnikov et al., 2022). This suggests that gastrointestinal diseases differed between South Asians and Africans due to differences in the gut microbiome (Donin et al., 2010; Porras et al., 2021).

**Fig. 3.**
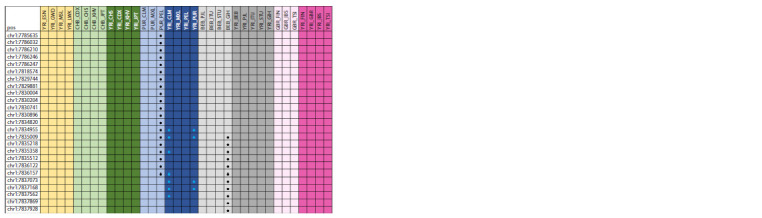
Genetic variants under selection pressure in the PER3 gene. Dots represent variants under selection pressure; black color of the dots means that the ancestral allele was under selection pressure, blue color stands for the derived allele.

This study identified 42 points of selection pressure in the PER3 gene when comparing East Asians living in Bangladesh (BEB) with East Asians living in the Houston, Texas area (GIH), mixed Americans living in Puerto Rico (PUR) with mixed Americans living in Peru (PEL), and when comparing Africans with YRI_CLM Colombians and Africans with YRI_PUR Puerto Ricans (Fig. 3). After annotation, the following associations with the SNPs found were identified: response to the use of lithium medications in the treatment of bipolar disorder, formation of chronotypes of different types, predisposition to depressive disorders, predisposition to metabolic syndrome, likelihood of developing colorectal cancer, and predisposition to obesity. More details of SNP associations with different diseases and phenotypes in humans are presented in Supplementary Material.

The main function of the sites identified by us as being under selection pressure was the formation of the morningtype chronotype. It is worth noting that the internal comparison of the groups of mixed Americans and East Asians identified ancestral alleles, while the comparison of Africans with mixed Americans identified derived alleles. Perhaps the key difference between Africans and mixed Americans is the sleep specificity of these populations. For example, mixed Americans are more likely to have an evening chronotype while Africans have the most frequent morning chronotype (Egan et al., 2017). This may be due to the influence of various external factors such as latitude, longitude, magnetic field action or solar activity.

In addition, the data obtained suggest the influence of external factors on the formation of the studied populations, which, as a result, led to different action of mechanisms of their adaptive abilities. For example, the isolation of the Lima- Callao mixed American (PEL) population from Africans may be due to the remoteness of location of this group of people compared to the other ethnic groups under study. It is reliably known that ethnicities from other parts of Latin America were subjected to more frequent mixing with Europeans compared to those from Peru (Chacón-Duque et al., 2018). Thus, the identity of the resulting population formed the most isolated genetic cluster.

## Discussion

Progressive statistical methods aimed at finding loci under selection pressure have allowed scientists from different countries to conduct studies on this topic. In the authors’ works, there are references to individual SNPs that we identified in this study as being under selection pressure. In total, we annotated 35 such sites

Researchers have done the work of annotating SNPs, finding association with polymorphisms at these sites and correlation with some diseases and physiological features. For example, S.E. Jones et al. analyzed behavioral indicators of circadian rhythms by analyzing whole-genome data in 697,828 residents of the United Kingdom (UK). The study uncovered novel loci associated with the morning-type chronotype. Among these loci, rs58574366 (2:238286196) was identified. Our analyses revealed that this SNP is under selection pressure in comparison groups of Europeans from the United Kingdom (GBR) with Europeans from Finland (FIN). The negative values of the xpehh calculation indices led us to conclude that derived alleles were detected in the two compared samples (Jones et al., 2019)

Another point of interest is rs74508725 (2:238278568). This outlier is found when comparing groups of Europeans from the United Kingdom (GBR) with Europeans from Finland (FIN) and carries negative values, which may indicate differentiation of this site within the studied groups. In the works of G. Kichaev and co-authors (Kichaev et al., 2019), this locus was associated with the phenotype expressed in participants’ height.

Locus rs2585399 (17:8151441) was identified by us as being under selection pressure when comparing several groups of people under study at once. These groups include comparisons of East Asians from Bangladesh (BEB) with East Asians from Texas (GIH) and South Asians from Beijing (CHB) with South Asians from Yunan (CDX). An interesting fact is that this SNP was associated with major depressive disorder in the authors’ study. Transcriptome association analysis revealed significant associations with NEGR1 expression in the hypothalamus and DRD2 expression in the contiguous nucleus (Levey et al., 2021).

Another selection signal studied previously was rs228654 (1:7837168). However, it is worth noting that comparisons between African and mixed American YRI (Ibadané, Nigeria) to CLM (Medellín, Colombia) and YRI (Ibadané, Nigeria) to PUR (Ruerto Rico) populations revealed negative EHH values, suggesting the presence of a derived allele between the groups. In contrast, positive selection values were found between the groups of East Asians living in Texas (BEB) and East Asians living in Bangladesh (GIH), indicating the presence of an ancestral allele. A group of researchers led by P.R. Jansen (Jansen et al., 2019) analyzed the human genome to gain insights into the pathways, tissues, and cell types involved in the regulation of insomnia. The single nucleotide polymorphism rs228654 was among the loci associated with the development of this disease.

## Conclusion

This study suggests that living conditions, climate, and other external factors directly influence the genetic structure of populations and vary by race and geographic location. In addition, many of the selection variants in the PER1, PER2, PER3 genes appear to regulate biological processes that are associated with major modern diseases including obesity, cancer, metabolic syndrome, bipolar personality disorder, depression, rheumatoid arthritis, diabetes mellitus, lupus erythematosus, stroke and Alzheimer’s disease, making them extremely interesting targets for further research aimed at identifying causal variants of human diseases, including cardiometabolic and psychiatric disorders, as well as cancer.

## Conflict of interest

The authors declare no conflict of interest.

## References

1000 Genomes. [WWW Document]. 2008. URL: https://www.ncbi.
nlm.nih.gov/projects/faspftp/1000genomes/ (accessed 9.6.23)

Ahrens C.W., Rymer P.D., Stow A., Bragg J., Dillon S., Umbers K.D.L.,
Dudaniec R.Y. The search for loci under selection: trends, biases
and progress. Mol. Ecol. 2018;27(6):1342-1356. DOI 10.1111/mec.
14549

Azevedo P.G., Miranda L.R., Nicolau E.S., Alves R.B., Bical-ho
M.A.C., Couto P.P., Ramos A.V., Souza R.P., Longhi R., Friedman
E., Marco L., Bastos-Rodrigues L. Genetic association of the
PERIOD3 (PER3) Clock gene with extreme obesity. Obes. Res.
Clin. Pract. 2021;15(4):334-338. DOI 10.1016/j.orcp.2021.06.006

Bacalini M.G., Palombo F., Garagnani P., Giuliani C., Fiorini C., Caporali
L., Stanzani Maserati M., Capellari S., Romagnoli M., De Fanti
S., Benussi L., Binetti G., Ghidoni R., Galimberti D., Scarpini E.,
Arcaro M., Bonanni E., Siciliano G., Maestri M., Guarnieri B.; Italian
Multicentric Group on clock genes, actigraphy in AD; Martucci
M., Monti D., Carelli V., Franceschi C., La Morgia C., Santoro A.
Association of rs3027178 polymorphism in the circadian clock gene
PER1 with susceptibility to Alzheimer’s disease and longevity in an
Italian population. GeroScience. 2022;44(2):881-896. DOI 10.1007/
s11357-021-00477-0

Bakoev S., Getmantseva L., Kostyunina O., Bakoev N., Prytkov Y.,
Usatov A., Tatarinova T.V. Genome-wide analysis of genetic diversity
and artificial selection in Large White pigs in Russia. PeerJ.
2021;9:e11595. DOI 10.7717/peerj.11595

Bakoev S.Y., Korobeinikova A.V., Mishina A.I., Kabieva S.S., Mitrofanov
S.I., Ivashechkin A.A., Akinshina A.I., Snigir E.A., Yudin
S.M.,
Yudin V.S., Getmantseva L.V., Anderzhanova E.A. Genomic signatures
of positive selection in human populations of the OXT, OXTR,
AVP, AVPR1A and AVR1B gene variants related to the regulation of
psychoemotional response. Genes (Basel). 2023;14(11): 2053. DOI
10.3390/genes14112053

Baranger D.A.A., Ifrah C., Prather A.A., Carey C.E., Corral-Frías N.S.,
Drabant Conley E., Hariri A.R., Bogdan R. PER1 rs3027172 genotype
interacts with early life stress to predict problematic alcohol
use, but not reward-related ventral striatum activity. Front. Psychol.
2016;7:464. DOI 10.3389/fpsyg.2016.00464

Benton M.L., Abraham A., LaBella A.L., Abbot P., Rokas A., Capra
J.A. The influence of evolutionary history on human health and
disease. Nat. Rev. Genet. 2021;22(5):269-283. DOI 10.1038/s41576-
020-00305-9

Biscontin A., Zarantonello L., Russo A., Costa R., Montagnese S.
Toward a molecular approach to chronotype assessment. J. Biol.
Rhythms. 2022;37(3):272-282. DOI 10.1177/07487304221099365

Blomeyer D., Buchmann A.F., Lascorz J., Zimmermann U.S., Esser G.,
Desrivieres S., Schmidt M.H., Banaschewski T., Schumann G.,
Laucht M. Association of PER2 genotype and stressful life events
with alcohol drinking in young adults. PLoS One. 2013;8(3):e59136.
DOI 10.1371/journal.pone.0059136

Bondarenko E.A., Shadrina M.I., Druzhkova T.A., Akzhigitov R.G.,
Gulyaeva N.V., Gekht A.B., Slominsky P.A. An association study
of rs10462021 polymorphism in the clock gene PERIOD3 and different
clinical types of depression. Mol. Genet. Microbiol. Virol. 2018;
33(1):26-29. DOI 10.3103/S0891416818010056

Cade B.E. Variation and selection in human circadian clock genes. Doctoral
Thesis. University of Surrey, 2010

Carlson C.S., Thomas D.J., Eberle M.A., Swanson J.E., Livingston
R.J., Rieder M.J., Nickerson D.A. Genomic regions exhibiting
positive selection identified from dense genotype data. Genome Res.
2005;15(11):1553-1565. DOI 10.1101/gr.4326505

Carpen J.D., Archer S.N., Skene D.J., Smits M., von Schantz M.
A single‐nucleotide polymorphism in the 5′‐untranslated region of
the hPER2 gene is associated with diurnal preference. J. Sleep Res.
2005;14(3):293-297. DOI 10.1111/j.1365-2869.2005.00471.x

Chacón-Duque J.-C., Adhikari K., Fuentes-Guajardo M., Mendoza-
Revilla J., Acuña-Alonzo V., Barquera R., Quinto-Sánchez M., …
Poletti G., Gallo C., Bedoya G., Rothhammer F., Balding D., Hellenthal
G., Ruiz-Linares A. Latin Americans show wide-spread
Converso ancestry and imprint of local Native ancestry on physical
appearance. Nat. Commun. 2018;9(1):5388. DOI 10.1038/s41467-
018-07748-z

Chang A.-M., Bjonnes A.C., Aeschbach D., Buxton O.M., Gooley
J.J.,
Anderson C., Van Reen E., Cain S.W., Czeisler C.A., Duffy J.F.,
Lockley S.W., Shea S.A., Scheer F.A.J.L., Saxena R. Circadian
gene variants influence sleep and the sleep electroencephalogram in
humans. Chronobiol. Int. 2016;33(5):561-573. DOI 10.3109/0742
0528.2016.1167078

Chang Y.-C., Chiu Y.-F., Liu P.-H., Hee S.W., Chang T.-J., Jiang Y.-D.,
Lee W.-J., Lee P.-C., Kao H.-Y., Hwang J.-J., Chuang L.-M. Genetic
variation in the NOC gene is associated with body mass index in chinese
subjects. PLoS One. 2013;8(7):e69622. DOI 10.1371/journal.
pone.0069622

Chappuis S., Ripperger J.A., Schnell A., Rando G., Jud C., Wahli W.,
Albrecht U. Role of the circadian clock gene Per2 in adaptation to
cold temperature. Mol. Metab. 2013;2(3):184-193. DOI 10.1016/
j.molmet.2013.05.002

Clemente J.C., Pehrsson E.C., Blaser M.J., Sandhu K., Gao Z., Wang B.,
Magris M., Hidalgo G., Contreras M., Noya-Alarcón Ó., Lander O.,
McDonald J., Cox M., Walter J., Oh P.L., Ruiz J.F., Rodriguez S.,
Shen N., Song S.J., Metcalf J., Knight R., Dantas G., Dominguez-
Bello M.G. The microbiome of uncontacted Amerindians. Sci. Adv.
2015;1(3):e1500183. DOI 10.1126/sciadv.1500183

Dan Y.-L., Zhao C.-N., Mao Y.-M., Wu Q., He Y.-S., Hu Y.-Q.,
Xiang K., Yang X.-K., Sam N.B., Wu G.-C., Pan H.-F. Association
of PER2 gene single nucleotide polymorphisms with genetic
susceptibility to systemic lupus erythematosus. Lupus. 2021;30(5):
734-740. DOI 10.1177/0961203321989794

Donin A.S., Nightingale C.M., Owen C.G., Rudnicka A.R., McNamara
M.C., Prynne C.J., Stephen A.M., Cook D.G., Whincup P.H.
Nutritional composition of the diets of South Asian, black African-
Caribbean and white European children in the United Kingdom:
The Child Heart and Health Study in England (CHASE). Br. J. Nutr.
2010;104(2):276-285. DOI 10.1017/S000711451000070X

Egan K.J., Knutson K.L., Pereira A.C., von Schantz M. The role of race
and ethnicity in sleep, circadian rhythms and cardiovascular health.
Sleep Med. Rev. 2017;33:70-78. DOI 10.1016/j.smrv.2016.05.004

Forbes E.E., Dahl R.E., Almeida J.R.C., Ferrell R.E., Nimgaonkar V.L.,
Mansour H., Sciarrillo S.R., Holm S.M., Rodriguez E.E., Phillips
M.L. PER2 rs2304672 polymorphism moderates circadianrelevant
reward circuitry activity in adolescents. Biol. Psychiatry.
2012;71(5):451-457. DOI 10.1016/j.biopsych.2011.10.012

Gafarov V.V., Gagulin I.V., Gromova E.A., Gafarova A.V., Panov
D.O.
Association of polymorphism rs934945 gene Per2 with sleep disorders
in the male population of Novosibirsk 25-44. Mir Nauki,
Kul’tury, Obrazovaniya = The World of Science, Culture and Education.
2016;5:283-287 (in Russian)

Gintis H., Doebeli M., Flack J. The evolution of human cooperation.
Cliodynamics. 2012;3(1):172-190. DOI 10.21237/C7CLIO3112928

Gu Z., Wang B., Zhang Y.-B., Ding H., Zhang Y., Yu J., Gu M., Chan P.,
Cai Y. Association of ARNTL and PER1 genes with Parkinson’s
disease: a case-control study of Han Chinese. Sci. Rep. 2015;5(1):
15891. DOI 10.1038/srep15891

Guo H., Li X., Li W., Wu J., Wang S., Wei J. Climatic modification effects
on the association between PM1 and lung cancer incidence in
China. BMC Public Health. 2021;21(1):880. DOI 10.1186/s12889-
021-10912-8

Hancock A.M., Alkorta-Aranburu G., Witonsky D.B., Di Rienzo A.
Adaptations to new environments in humans: the role of subtle
allele frequency shifts. Philos. Trans. R. Soc. B Biol. Sci. 2010;
365(1552):2459-2468. DOI 10.1098/rstb.2010.0032

Holipah Hinoura T., Kozaka N., Kuroda Y. The correlation between
PER3 rs2640908 polymorphism and colorectal Cancer in the Japanese
population. Appl. Cancer Res. 2019;39(1):3. DOI 10.1186/
s41241-019-0072-5

Jansen P.R., Watanabe K., Stringer S., Skene N., Bryois J., Hammerschlag
A.R., de Leeuw C.A., Benjamins J.S., Muñoz-Manchado
A.B., Nagel M., Savage J.E., Tiemeier H., White T., Tung J.Y.,
Hinds D.A., Vacic V., Wang X., Sullivan P.F., van der Sluis S., Polderman
T.J.C., Smit A.B., Hjerling-Leffler J., Van Someren E.J.W.,
Posthuma D. Genome-wide analysis of insomnia in 1,331,010 individuals
identifies new risk loci and functional pathways. Nat. Genet.
2019;51(3):394-403. DOI 10.1038/s41588-018-0333-3

Jensen J.D., Foll M., Bernatchez L. The past, present and future of genomic
scans for selection. Mol. Ecol. 2016;25(1):1-4. DOI 10.1111/
mec.13493

Jones S.E., Lane J.M., Wood A.R., van Hees V.T., Tyrrell J., Beaumont
R.N., Jeffries A.R., … Gehrman P.R., Lawlor D.A., Frayling
T.M., Rutter M.K., Hinds D.A., Saxena R., Weedon M.N.
Genome-wide association analyses of chronotype in 697,828 individuals
provides insights into circadian rhythms. Nat. Commun.
2019;10(1):343. DOI 10.1038/s41467-018-08259-7

Kichaev G., Bhatia G., Loh P.-R., Gazal S., Burch K., Freund M.K.,
Schoech A., Pasaniuc B., Price A.L. Leveraging polygenic functional
enrichment to improve GWAS power. Am. J. Hum. Genet.
2019;104(1):65-75. DOI 10.1016/j.ajhg.2018.11.008

Kim J.-W., Kim H.-A., Suh C.-H., Jung J.-Y. Sex hormones affect the
pathogenesis and clinical characteristics of systemic lupus erythematosus.
Front. Med. 2022;9:906475. DOI 10.3389/fmed.2022.
906475

Klassmann A., Gautier M. Detecting selection using extended haplotype
homozygosity (EHH)-based statistics in unphased or unpolarized
data. PLoS One. 2022;17(1):e0262024. DOI 10.1371/journal.
pone.0262024

Kripke D.F., Nievergelt C.M., Joo E., Shekhtman T., Kelsoe J.R. Circadian
polymorphisms associated with affective disorders. J. Circadian
Rhythms. 2009;7:2. DOI 10.1186/1740-3391-7-2

Lee H., Nah S.-S., Chang S.-H., Kim H.-K., Kwon J.-T., Lee S.,
Cho I.- H., Lee S.W., Kim Y.O., Hong S.-J., Kim H.-J. PER2 is
downregulated by the LPS-induced inflammatory response in synoviocytes
in rheumatoid arthritis and is implicated in disease susceptibility.
Mol. Med. Rep. 2017;16(1):422-428. DOI 10.3892/mmr.
2017.6578

Lesicka M., Jabłońska E., Wieczorek E., Pepłońska B., Gromadzińska
J., Seroczyńska B., Kalinowski L., Skokowski J., Reszka E.
Circadian gene polymorphisms associated with breast cancer susceptibility.
Int. J. Mol. Sci. 2019;20(22):5704. DOI 10.3390/ijms
20225704

LeVan T.D., Xiao P., Kumar G., Kupzyk K., Qiu F., Klinkebiel D.,
Eudy J., Cowan K., Berger A.M. Genetic variants in circadian
rhythm genes and self-reported sleep quality in women with breast
cancer. J. Circadian Rhythms. 2019;17(1):184. DOI 10.5334/
jcr.184

Levey D.F., Stein M.B., Wendt F.R., Pathak G.A., Zhou H., Aslan M.,
Quaden R., Harrington K.M., Nuñez Y.Z., Overstreet C., Radhakrishnan
K., Sanacora G., McIntosh A.M., Shi J., Shringarpure S.S.,
Concato J., Polimanti R., Gelernter J. Bi-ancestral depression GWAS
in the Million Veteran Program and meta-analysis in >1.2 million individuals
highlight new therapeutic directions. Nat. Neurosci. 2021;
24(7):954-963. DOI 10.1038/s41593-021-00860-2

Levran O., Randesi M., Rotrosen J., Ott J., Adelson M., Kreek M.J.
A 3′ UTR SNP rs885863, a cis-eQTL for the circadian gene VIPR2
and lincRNA 689, is associated with opioid addiction. PLoS One.
2019;14(11):e0224399. DOI 10.1371/journal.pone.0224399

Liberman A.R., Kwon S.B., Vu H.T., Filipowicz A., Ay A., Ingram K.K.
Circadian clock model supports molecular link between PER3 and
human anxiety. Sci. Rep. 2017;7(1):9893. DOI 10.1038/s41598-017-
07957-4

Lin E., Kuo P.-H., Liu Y.-L., Yang A.C., Kao C.-F., Tsai S.-J. Effects of
circadian clock genes and health-related behavior on metabolic syndrome
in a Taiwanese population: Evidence from association and interaction
analysis. PLoS One. 2017;12(3):e0173861. DOI 10.1371/
journal.pone.0173861

Lopez M.E., Neira R., Yáñez J.M. Applications in the search for genomic
selection signatures in fish. Front. Genet. 2015;5:458. DOI
10.3389/fgene.2014.00458

Maryanaji Z. The effect of climatic and geographical factors on breast
cancer in Iran. BMC Res. Notes. 2020;13(1):519. DOI 10.1186/
s13104-020-05368-9

McCarthy M.J., Welsh D.K. Cellular circadian clocks in mood disorders.
J. Biol. Rhythms. 2012;27(5):339-352. DOI 10.1177/0748730
412456367

Melhuish Beaupre L.M., Gonçalves V.F., Zai C.C., Tiwari A.K., Harripaul
R.S., Herbert D., Freeman N., Müller D.J., Kennedy J.L.
Genome-wide association study of sleep disturbances in depressive
disorders. Mol. Neuropsychiatry. 2020;5(Suppl. 1):34-43. DOI
10.1159/000505804

Min W., Tang N., Zou Z., Chen Y., Zhang X., Huang Y., Wang J.,
Zhang Y., Zhou B., Sun X. A panel of rhythm gene polymorphisms
is involved in susceptibility to type 2 diabetes mellitus and bipolar
disorder. Ann. Transl. Med. 2021;9(20):1555. DOI 10.21037/atm-
21-4803

Miranda A., Shekhtman T., McCarthy M., DeModena A., Leckband
S.G., Kelsoe J.R. Study of 45 candidate genes suggests
CACNG2 may be associated with lithium response in bipolar disorder.
J. Affect. Disord. 2019;248:175-179. DOI 10.1016/j.jad.2019.
01.010

Molina E., Gould N., Lee K., Krimins R., Hardenbergh D., Timlin H.
Stress, mindfulness, and systemic lupus erythematosus: An overview
and directions for future research. Lupus. 2022;31(13):1549-
1562. DOI 10.1177/09612033221122980

National Library of Medicine (US) [WWW Document]. URL https://
www.ncbi.nlm.nih.gov/(accessed 9.17.23)

Nielsen R. Molecular signatures of natural selection. Annu. Rev. Genet.
2005;39(1):197-218. DOI 10.1146/annurev.genet.39.073003.
112420

Pan Z., Yu L., Shao M., Ma Y., Cheng Y., Wu Y., Xu S., Zhang C.,
Zhu J., Pan F., Sun G. The influence of meteorological factors and
total malignant tumor health risk in Wuhu city in the context of climate
change. BMC Public Health. 2023;23(1):346. DOI 10.1186/
s12889-023-15200-1

Porras A.M., Shi Q., Zhou H., Callahan R., Montenegro-Bethancourt G.,
Solomons N., Brito I.L. Geographic differences in gut microbiota
composition impact susceptibility to enteric infection. Cell Rep.
2021;36(4):109457. DOI 10.1016/j.celrep.2021.109457

Purcell S., Neale B., Todd-Brown K., Thomas L., Ferreira M.A.R.,
Bender D., Maller J., Sklar P., de Bakker P.I.W., Daly M.J., Sham P.C.
PLINK: A tool set for whole-genome association and populationbased
linkage analyses. Am. J. Hum. Genet. 2007;81(3):559-575.
DOI 10.1086/519795

Qu F., Qiao Q., Wang N., Ji G., Zhao H., He L., Wang H., Bao G.
Genetic
polymorphisms in circadian negative feedback regulation
genes predict overall survival and response to chemotherapy in gastric
cancer patients. Sci. Rep. 2016;6(1):22424. DOI 10.1038/srep
22424

Quaglia M., Merlotti G., De Andrea M., Borgogna C., Cantaluppi V.
Viral infections and systemic lupus erythematosus: new players in
an old story. Viruses. 2021;13(2):277. DOI 10.3390/v13020277

Rijo-Ferreira F., Takahashi J.S. Genomics of circadian rhythms in
health and disease. Genome Med. 2019;11(1):82. DOI 10.1186/
s13073-019-0704-0

Sabeti P.C., Reich D.E., Higgins J.M., Levine H.Z.P., Richter D.J.,
Schaffner S.F., Gabriel S.B., Platko J.V., Patterson N.J., McDonald
G.J., Ackerman H.C., Campbell S.J., Altshuler D., Cooper R.,
Kwiatkowski D., Ward R., Lander E.S. Detecting recent positive
selection in the human genome from haplotype structure. Nature.
2002;419(6909):832-837. DOI 10.1038/nature01140

Sakurada K., Konta T., Takahashi S., Murakami N., Sato H., Murakami
R., Watanabe M., Ishizawa K., Ueno Y., Yamashita H., Kayama T.
Circadian clock gene polymorphisms and sleep-onset problems in a
population-based cohort study: The yamagata study. Tohoku J. Exp.
Med. 2021;255(4):325-331. DOI 10.1620/tjem.255.325

Saravanan K.A., Panigrahi M., Kumar H., Bhushan B., Dutt T.,
Mishra B.P. Selection signatures in livestock genome: A review
of concepts, approaches and applications. Livest. Sci. 2020;241:
104257. DOI 10.1016/j.livsci.2020.104257

Schroor M.M., Plat J., Mensink R.P. Relation between single nucleotide
polymorphisms in circadian clock relevant genes and cholesterol
metabolism. Mol. Genet. Metab. 2023;138(4):107561. DOI
10.1016/j.ymgme.2023.107561

Shareefa D. Genetic analysis of bipolar disorder and alcohol use disorder.
Doctoral Thesis. University of Cape Town, 2015

Shi Y., Liu Y., Yang L., Yan J. A mathematical model to characterize the
role of light adaptation in mammalian circadian clock. Front. Mol.
Biosci. 2021;8:681696. DOI 10.3389/fmolb.2021.681696

Soria V., Martínez-Amorós È., Escaramís G., Valero J., Pérez-Egea R.,
García C., Gutiérrez-Zotes A., Puigdemont D., Bayés M., Crespo
J.M., Martorell L., Vilella E., Labad A., Vallejo J., Pérez V.,
Menchón J.M., Estivill X., Gratacòs M., Urretavizcaya M. Differential
association of circadian genes with mood disorders: CRY1 and
NPAS2 are associated with unipolar major depression and CLOCK
and VIP with bipolar disorder. Neuropsychopharmacology. 2010;
35(6):1279-1289. DOI 10.1038/npp.2009.230

Syromyatnikov M., Nesterova E., Gladkikh M., Smirnova Y., Gryaznova
M., Popov V. Characteristics of the gut bacterial composition
in people of different nationalities and religions. Microorganisms.
2022;10(9):1866. DOI 10.3390/microorganisms10091866

Szpiech Z.A. Selscan 2.0: scanning for sweeps in unphased data.
bioRxiv.
2021. DOI 10.1101/2021.10.22.465497

Voight B.F., Kudaravalli S., Wen X., Pritchard J.K. A map of recent
positive selection in the human genome. PLoS Biol. 2006;4(3):e72.
DOI 10.1371/journal.pbio.0040072

Wang W.M., Yuan P., Wang J.Y., Ma F., Fan Y., Li Q., Zhang P., Xu B.H.
Association of genetic variantions of circadian clock genes and risk of breast cancer. Zhonghua Zhong Liu Za Zhi. 2013;35(3):236-239.
DOI 10.3760/cma.j.issn.0253-3766.2013.03.017

Wen M., Jiang X., She H., Han C., Pei Z., Cai Y., Zhang T. The Per2
polymorphism rs10462023 is associated with the risk of stroke in
a Chinese population. Biol. Rhythm Res. 2015;46(4):545-551. DOI
10.1080/09291016.2015.1026675

Xu L., Bickhart D.M., Cole J.B., Schroeder S.G., Song J., Tassell C.P.,
Sonstegard T.S., Liu G.E. Genomic signatures reveal new evidences
for selection of important traits in domestic cattle. Mol. Biol. Evol.
2015;32(3):711-725. DOI 10.1093/molbev/msu333

Zhang L., Ptáček L.J., Fu Y.-H. Diversity of human clock genotypes and
consequences. 2013;119:51-81. DOI 10.1016/B978-0-12-396971-
2.00003-8

Zheng W., He Y., Guo Y., Yue T., Zhang H., Li J., Zhou B., Zeng X.,
Li L., Wang B., Cao J., Chen L., Li C., Li H., Cui C., Bai C., Baimakangzhuo,
Qi X., Ouzhuluobu, Su B. Large-scale genome sequencing
redefines the genetic footprints of high-altitude adaptation in
Tibetans. Genome Biol. 2023;24(1):73. DOI 10.1186/s13059-023-
02912-1

